# Transglutaminase Cross-Linked and Lysozyme-Incorporated Antimicrobial Tilapia Collagen Edible Films: Development and Characterization

**DOI:** 10.3390/foods12071475

**Published:** 2023-03-30

**Authors:** Bing-Yi Hou, Be-Jen Wang, Yih-Ming Weng

**Affiliations:** Department of Food Science, National Chiayi University, 300 University Road, Chiayi 60004, Taiwan

**Keywords:** tilapia collagen, lysozyme, transglutaminase, antimicrobial packaging

## Abstract

To improve the mechanical properties and confer antimicrobial activity, transglutaminase (TGase) was used as a cross-linking agent and lysozyme (LYS) was incorporated as an antimicrobial agent to prepare novel active tilapia collagen (TC) films. While the difference in visual appearance was not obvious, the LYS incorporation increased the opacity of TC films. The water vapor permeability of all TGase cross-linked TC films was significantly (*p* < 0.05) lower than that of the control film (prepared without TGase and LYS). In addition, while the tensile strength and Young’s modulus of all TGase cross-linked TC films were significantly (*p* < 0.05) higher than those of the control film, elongation at break of all TGase cross-linked TC films was significantly (*p* < 0.05) lower than that of the control film. LYS incorporated TC films showed antimicrobial activity against *E. coli*, *Staphylococcus aureus*, *Enterococcus faecium*, *Bacillus subtilis* and *Pseudomonas fluorescens.* Collectively, TC films with improved physiochemical properties and antimicrobial activity have a good potential to serve as active food packaging materials.

## 1. Introduction

The functions of food packaging include protecting food from physiochemical degradation, preventing microbial contamination, and providing information to consumers about product content and storage conditions [1]. In addition to relatively low cost, petroleum-based synthetic plastics are the most commonly used food packaging materials because of barrier and mechanical properties [2]. However, almost all synthetic plastics are non-recyclable and non-biodegradable, and plastic waste has caused serious environmental problems [3]. As a possible alternative, biodegradable packaging materials such as edible films and coatings are actively being developed for the possible replacement of synthetic plastics in order to reduce environmental pollution arising from incineration or landfill of synthetic materials [4].

Biomaterials commonly used for the preparation of edible films and coatings are polysaccharides, proteins and lipids. Polysaccharides include chitosan, starch, cellulose, pectin, alginate, etc. Proteins include collagen, casein, whey protein, pea protein, maize zein, etc. Lipids include waxes, vegetable-based oils, fatty acids, etc. Among different categories, proteins are suitable candidates for possessing various beneficial physicochemical functionalities [5]. Proteins are polymers of amino acids and generally have good film-forming ability and good nutritional value. After protein is denatured using acid, alkaline, solvent or heating to extend the molecular chains, protein films are formed via hydrogen bonds, ionic bonds or covalent bonds through interacting with neighboring molecules. Protein films generally have good gas and aroma barrier properties, and their mechanical properties are also better than films made of polysaccharides and lipids.

As a special type of protein, collagen is widely present in various tissues and organs of animals. Collagen, composed of three identical or slightly different polypeptide chains forming a coiled triple-helix structure, has good biocompatibility and film-forming properties. Currently, the main sources of commercial collagen are the skin and bone of cattle and swine [6]. Due to concerns about transmissible diseases such as bovine spongiform encephalopathy (BSE) and religious constraints, fish collagen has feasible and promising potential for serving as edible packaging materials [7,8]. Tilapia (*Oreochromis niloticus*) is an important aqua-cultural fish species in tropical, subtropical and temperate regions around the world [9]. It was estimated that about 60–70% of body mass (including bone, skin, scale, head and internal organs) ended up as waste during the processing of tilapia [10]. Since fish skin and scale are rich in collagen [11], the valorization and application of tilapia waste are worth investigating.

However, relatively high hygroscopicity and the resulting insufficient tensile strength are major disadvantages of films made of collagen. Cross-linking reaction among protein molecules can be used as an alternative strategy to improve the mechanical strength and barrier properties of protein films [6,12]. Through forming intermolecular or intramolecular covalent or non-covalent bonds among collagen molecules, physical, chemical or enzymatic cross-linking reactions have been used to improve the physicochemical and mechanical properties of collagen films [13,14]. Among various methods, enzymatic cross-linking possesses the best feasibility for safety reason.

Transglutaminase (TGase) catalyzes the formation of an intermolecular or intramolecular ε-(γ-glutaminyl)-lysine covalent bond between the γ-carboxamide group of glutamic acid and the ε-amino group of lysine [15,16]. Applications of TGase to improve the physicochemical properties of proteins are found in the literature. TGase increased the resilience and strength of dough [17]. TGase was used in restructured meat via reactions with actin and myosin to form Gln-Lys isopeptide bonds, which improved the texture and gel strength of meat products during freezing, refrigeration or cooking [16,18]. TGase improved the mechanical properties and thermal-stability properties of the collagen films [14]. Water vapor permeability of whey protein/pectin composite films applied on French fries and donuts was decreased by utilizing TGase as the cross-linking agent [19]. Ahammed et al. prepared gelatin-zein composite membranes with TGase as a cross-linking agent and found that TGase cross-linking could enhance the mechanical properties of the films and reduce their water solubility [20].

To extend the shelf life and improve the safety of foods, substances can be effectively released into or absorbed from packaged food by active food packaging materials [21]. Lysozyme (LYS) from egg protein has the GRAS status conferred by the US FDA [22]. The antimicrobial activity of lysozyme is mainly achieved by hydrolyzing the β-(1,4)-glycosidic bond between N-acetylmuramic acid and N-acetylglucosamine of peptidoglycan in the bacterial cell wall [23,24]. Antimicrobial edible films have been fabricated with LYS [25]. The total bacterial count and lipid oxidation of fresh fish fillets were reduced using chitosan coatings incorporated with LYS [26].

Since research on simultaneously improving the physiochemical properties and conferring the antimicrobial activities of TC films are rarely found in the literature, the objectives of the present study were to fabricate TGase cross-linked TC films with antimicrobial activity via incorporation with LYS. The physiochemical properties and antibacterial ability of the resulting films were determined to demonstrate the feasibility of these PC films to serve as novel active packaging materials.

## 2. Materials and Methods

### 2.1. Preparation of Tilapia Collagen Films

Tilapia collagen (TC) films were prepared according to [27] with modifications. Tilapia collagen (2.4 g; Nitta Gelatin, Morrisville, NC, USA) and deionized water (20 mL) were stirred at 60 °C for 30 min. After adding glycerol (0.6 g; Sigma-Aldrich, St. Louis, MO, USA) and stirring for another 30 min, the pH was adjusted to 6.7 with 0.1 M NaOH. Transglutaminase (TGase, 72 mg, *w*/*w* TC, 80–130 units/g; Micro-Tech Foods Ingredients, New Taipei City, Taiwan) was added into the film-forming mixture, which was then placed in a reciprocal water bath at 45 °C for 60 min. Then, TGase was inactivated at 80 °C for 10 min. After cooling to room temperature, lysozyme (LYS) at the levels of 0, 2, 4, 6, and 8 mg/g TC were added and mixed for an additional 30 min. Finally, the film forming mixture was poured onto a polystyrene petri dish (15 cm, dia.) and dried at 25 °C for 24 h. The resulting films were coded as TC-TGase-LYS-0, TC-TGase-LYS-2, TC-TGase-LYS-4, TC-TGase-LYS-6, and TC-TGase-LYS-8. Films were conditioned at 25 °C and 50% RH for 48 h before physicochemical analyses. The control film with only TC and glycerol was also prepared via the same procedure and used for comparison.

### 2.2. Optical Properties of TC Films

The color attributes (CIE L*, a* and b*) of film were measured with a color meter (NE4000, Nippon Denshoku Industries, Tokyo, Japan). L*, a* and b* represent the brightness/darkness, redness/greenness and yellowness/blueness of the sample, respectively [20]. Before measurement, a standard white tile (Y = 93.06, X = 95.06, Z = 112.04) was used for calibration using a 30 φ lens and a 30 φ colorimetric stage. The film sample was cut into a 3 × 3 cm square and fixed on the measurement stage. Under the transmission mode, the color attributes of each piece of film were the average of 5 randomly selected spots. Triplicate samples were used for each type of film. The whiteness index (WI) was calculated using Equation (1).

WI = 100 − [(100 − L)^2^ + (a*)^2^ + (b*)^2^]^1/2^ (1)

The opacity of the film was measured according to [28] with modifications. A film strip with a dimension of 1 × 4 cm was fixed on the inner wall of a quartz cuvette. The absorbance at 600 nm was measured. The opacity was calculated using Equation (2) and a higher value represented higher opacity. Triplicate samples were used for each type of film.

(2) $$\mathrm{Opacity}=\frac{{\mathrm{ABS}}_{600}}{X}$$

ABS_600_: the absorbance at 600 nm

X: the film thickness (mm)

### 2.3. Thickness, Moisture Content and Water Solubility of TC Films

The film thickness was measured with a micrometer (SM-114, Mitutoyo, Okaya, Japan). The thickness of each piece of film was the average of ten randomly selected spots. Triplicate samples were used for each type of film.

The moisture content was determined according to [2] with modifications. Briefly, film was heated at 105 °C until constant weight. The moisture content was calculated and expressed as g/100 g. Triplicate samples were used for each type of film.

The water solubility was determined according to [2] with some modifications. Film was cut into 3 × 3 cm piece (ca. 0.2 g). Each piece of film specimen was weighed (W_0_), placed in 15 mL distilled water and shaken (100 rpm) at 25 °C for 24 h. An insoluble portion was collected via filtration (Whatman #1 filter paper), rinsed with deionized water, dried at 105 °C for 24 h and weighed (W_1_) again. Triplicate samples were used for each type of film and the water solubility was calculated using Equation (3).

Water solubility (%) = [ (W_0_ − W_1_)/W_0_] × 100% (3)

### 2.4. Water Vapor Permeability (WVP) of TC Films

The water vapor permeability was determined according to [29] with modifications. A film sample and water vapor permeation device were conditioned at 25 °C and 65% RH for 24 h. After placing 70 g anhydrous silica gel into the device to achieve 0% RH inside the device, the film was mounted and sealed on the device rim. The whole device was weighed and then placed in a humidity chamber with 65% RH at 25 °C. Then, the device was weighed every 2 h for first 12 h and also weighed at 24 and 48 h.

### 2.5. Mechanical Properties of TG Films

Mechanical properties were determined according to [30] with minor modifications. Film specimens with dimensions of 50 × 10 mm were conditioned at 25 °C and 65% RH for 48 h. The film strip was mounted with the grips of a universal testing machine (AI-2500, Gotech, Taichung, Taiwan). The testing conditions were set as initial grip length 30 mm and crosshead speed 60 mm/min. While tensile strength and elongation at break were reported, Young’s modulus was calculated from the stress-strain curve.

### 2.6. Fourier-Transform Infrared Spectroscopy (FTIR) Analysis of TC Films

FTIR analysis was performed using a Fourier-transform infrared spectroscope (Spectrum 100, PerkinElmer, Inc., Waltham, IL, USA) equipped with an attenuated total reflectance (ATR) module attached to a ZnSe crystal. The film was placed on the ATR accessory and analyzed under transmission mode with the operation conditions as follows: scanning wavenumber 400–4000 cm^−1^, 32 scans and resolution 4 cm^−1^.

### 2.7. The Preparation of Testing Bacterial Cultures

*E. coli* (LYC 1656), *Staphylococcus aureus* (LYC 1659) and *Enterococcus faecium* (LYC 1513) were kindly provided by Dr. Lu Ying Chen, Department of Food Science, National Chiayi University (Chiayi, Taiwan). *Bacillus subtilis* (BCRC 10029) and *Pseudomonas fluorescens* (BCRC 11028) were purchased from the Bioresource Collection and Research Center (Hsinchu, Taiwan). Nutrient broth (NB) and nutrient agar (NA) were obtained from Merck (Darmstadt, Germany) and used for the cultivation of *E. coli* (LYC 1656), *S. aureus* (LYC 1659), *B. subtilis* (BCRC 10029) and *P. fluorescens* (BCRC 11028). MRS broth and MRS agar were purchased from HiMedia Laboratories (Dindhori, Nashik, India) and used for the cultivation of *E. faecium* (LYC 1513). While the incubation time was 24 h for all bacterial strains, the cultivation temperatures for *E. coli* (LYC 1656), *S. aureus* (LYC 1659), *B. subtilis* (BCRC 10029), *P. fluorescens* (BCRC 11028) and *E. faecium* (LYC 1513) were 37, 37, 30, 26 and 37 °C, respectively.

### 2.8. The Determination of Antimicrobial Activity of TC Films

For each bacterial strain, pure culture from a single colony on an agar plate was transferred into sterile broth medium. The overnight culture was obtained via incubation at the specific temperature mentioned above for 16–18 h and dilution with sterile broth medium to 10^3^–10^4^ CFU/mL for antimicrobial testing. The antibacterial activity of films was determined according to [31]. A piece of film (5 × 5 cm) was added into the mixture of 0.5 mL bacteria culture and 4.5 mL sterile broth medium. The testing mixture was incubated at a specified temperature with shaking for 24 h to determine the antimicrobial activity under dynamic contact conditions. An aliquot of 0.1 mL was sampled and diluted with fresh broth medium and spread on agar plates. Control (containing only 0.5 mL bacteria culture and 4.5 mL sterile broth medium) without film was tested under the same experimental protocol for the inhibition percentage calculation. After the plates were incubated at the specified temperature for 24 h, the colonies were counted and the inhibition percentage was calculated using Equation (4).

Inhibitory percentage (%) = [(CFU_control_ − CFU_sample_)/CFU_control_] × 100% (4)

CFU_control_: bacterial count of sample without film (CFU/mL)

CFU_sample_: bacterial count of sample with film (CFU/mL)

### 2.9. Statistical Analysis

The results were expressed as mean ± standard deviation of at least triplicate samples unless otherwise mentioned. Data were analyzed using SPSS software (Statistics Product for Service Solution, 20.0; International Business Machines Corporation, Armonk, NY, USA). One-way ANOVA and Duncan’s multiple range test were used to analyze the difference among samples. Differences were considered statistically significant when *p* < 0.05.

## 3. Result and Discussion

### 3.1. The Optical Properties of TC Films

**Figure 1:**
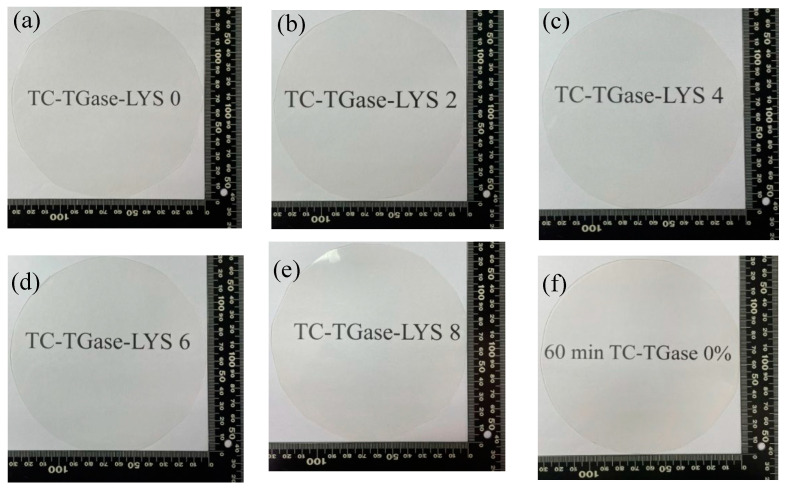
Photographs of transglutaminase (TGase) cross-linked tilapia collagen (TC) films incorporated with lysozyme (LYS). (**a**) TC-TGase-LYS-0, (**b**) TC-TGase-LYS-2, (**c**) TC-TGase-LYS-4, (**d**) TC-TGase-LYS-6, (**e**) TC-TGase-LYS-8 and (**f**) control film.

The appearance of the control film (without TAase and LYS) as well as films cross-linked using TGase and incorporated with different concentrations of LYS is shown in Figure 1. It appeared that all types of film are transparent with a smooth surface. There was no marked difference in appearance via visual inspection, indicating that the TGase cross-linking and LYS addition levels used in this study did not obviously affect the appearance of the films.

The results of instrumental color analysis of TC films are shown in Table 1. While the L* value of the control film was 95.27 ± 0.14, the cross-linking via TGase significantly (*p* < 0.05) lowered the L* as observed via the TC-TGase-LYS-0 film with 92.96 ± 0.09. However, the L* values for films with LYS were between 95.29 ± 0.02 and 95.37 ± 0.06, indicating that the incorporation of LYS increased the L*. Similar results were also obtained for WI. On the other hand, TC-TGase-LYS-0 film showed the lowest a* value (−0.28 ± 0.01) as compared with all other types of films. However, the b* values were not significantly affected by the TGase cross-linking and LYS incorporation. Similar results were reported in the literature [32]. The addition of LYS into carboxymethylcellulose films increased the L* and a* values, while there were no significant effects on the b* values.

While the opacity values of the control film and TC-TGase-LYS-0 were 0.30 ± 0.01 and 0.31 ± 0.01, respectively; the opacity of films containing LYS was in the range of 0.41 ± 0.01 to 0.42 ± 0.02 (Table 1). Since the higher opacity value indicated lower light transmittance, the incorporation of LYS could reduce light-induced food deterioration [33]. Similar results were found in the literature. The opacity of wheat starch edible films was increased by 13–15% with the addition of LYS [34].

### 3.2. The Thickness, Moisture Content and Water Solubility of TC Films

While the thickness of control film was 0.151 ± 0.004 mm, the thickness of the TGase cross-linked films increased with the increase of LYS content (Table 2). The thickness of film was affected by the total solid of the film-forming solution [35]. Therefore, TC-TGase-LYS-8 had the highest thickness value of 0.159 ± 0.003 mm. Bonomo et al. [36] reported that the thickness (0.14 to 0.17 mm) of the film was proportional to the content of LYS (0–10 g/100 g of starch) used to prepared the jackfruit starch edible films.

The moisture contents of films are shown in Table 2. While the moisture content of the control film was 13.76 ± 0.15 g/100 g, the moisture contents for TGase cross-linked films were in the range of 12.74 ± 0.16 to 12.02 ± 0.16 g/100 g. The results indicate that a tighter molecular structure via cross-linking might reduce the moisture content. Regarding the effects of LYS incorporation, the moisture content of TC-TGase-LYS-0 was significantly higher than those of films containing LYS. A similar report was found in the literature [37]. A lower moisture content of LYS-chitosan composite films was detected with the incorporation of LYS. The main reason might be that the molecular structure of LYS contains both hydrophobic and hydrophilic amino acids. During the film-forming process, the hydrophilic amino acid side chain of LYS would interact with the film-forming solution to form a hydrophobic core. Therefore, the addition of LYS increased the hydrophobic side chains in the membrane matrix and reduced the moisture content of the membrane.

The solubility of the edible film can be used as the suitability for packaging high moisture foods and an important factor in determining whether the packaging material is biodegradable [22]. After soaking about 0.2 g film in 15 mL distilled water for 24 h with shaking, the water solubility values of control film and TC-TGase-LYS-0 were 98.75 ± 1.38 and 98.87 ± 0.13%, respectively (Table 2). Tested with the same protocol, slightly lower water solubility ranges of 95.18 ± 1.04 to 96.59 ± 0.83% for films containing LYS were obtained. Furthermore, intermolecular interactions of proteins (hydrogen bonding and hydrophobic interactions) reduced protein–water interactions in solution, thereby reducing the solubility of films [38].

### 3.3. The Water Vapor Permeability TC Films

While the water vapor permeability of control film was 4.48 ± 0.19 × 10^−10^ g m/m^2^ s Pa (Table 2), significantly lower water vapor permeability was detected for TC-TGase-LYS-0 (1.97 ± 0.02 × 10^−10^ g m/m^2^ s Pa). The results indicated that cross-linking markedly reduced the migration of water molecules through the film matrix. However, the incorporation of LYS into film significantly increased the water vapor permeability. The water vapor permeability values of films containing LYS were in the range of 3.33 ± 0.18 to 3.84 ± 0.16 (10^−10^ g m/m^2^ s Pa). When LYS was added into fish skin gelatin film, the water vapor permeability of the film increased [39]. The addition of LYS would affect the binding affinity of the film matrix to water molecules, resulting in a decrease in the overall gel strength and water vapor barrier of the film. The addition of lysozyme would decrease the intermolecular attraction forces, increase the free volume between molecules and then increase the mobility of the bonds (consequently the mobility of the chains), resulting in the increase of water vapor penetration rate in the film matrix [36].

### 3.4. Mechanical Properties of TC Films

As compared with TGase cross-linked films, the control film had significantly (*p* < 0.05) lower tensile strength, elongation at break and Young’s modulus (Table 3). The results indicated that cross-linking with TGase markedly affected the mechanical properties of films. Regarding the films cross-linked with TGase, while the elongation at break decreased, the tensile strength and Young’s modulus of the film gradually increased as the LYS content increased. TGase-LYS-8 had the highest tensile strength of 3.07 ± 0.46 MPa and Young’s modulus of 384.77 ± 23.14 MPa. The formation of additional hydrogen bonds between amine groups and carboxyl groups increased the tensile strength of the egg protein and pullulan composite film [40]. Therefore, it can be speculated that amine groups and carboxyl groups in LYS would form more intermolecular hydrogen bonds with the hydroxyl group of TC and increase the tensile strength and Young’s modulus of films. When lysozyme was added into the gliadin film cross-linked with cinnamaldehyde, the tensile strength of the film increased and the elongation at break decreased [41].

### 3.5. FTIR Analysis of TC Films

**Figure 2:**
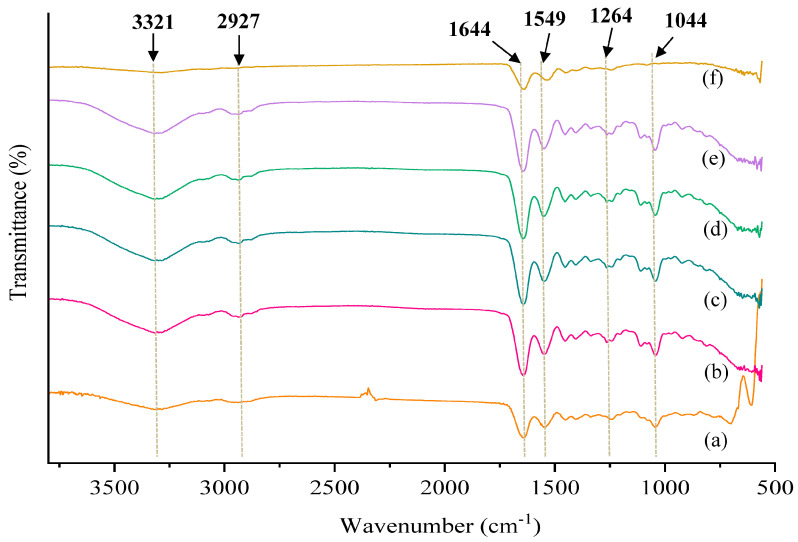
FTIR−ATR spectra of transglutaminase (TGase) cross-linked tilapia collagen (TC) films incorporated with lysozyme (LYS). (**a**) TC-TGase-LYS-0, (**b**) TC-TGase-LYS-2, (**c**) TC-TGase-LYS-4, (**d**) TC-TGase-LYS-6, (**e**) TC-TGase-LYS-8 and (**f**) Lysozyme.

The FTIR-ATR spectra of films are shown in Figure 2. The absorption peaks at 1644 and 1549 cm^−1^ are characteristic peaks of LYS, which are the C=O stretching vibration of amide I and N-H bending vibration and C-N stretching of amide II, respectively. Since the peak intensity of amide II (1500–1600 cm^−1^) is proportional to the amount of LYS [42], the intensities of peaks at 1644 and 1549 cm^−1^ markedly enhanced as the amount of LYS increased. The absorption peak appeared at 1264 cm^−1^, indicating that lysozyme formed an intermolecular force with the protein to form amide III (Hu et al., 2022). A new characteristic peak appeared at 1264 cm^−1^, representing a larger intermolecular force between lysozyme and collagen [32]. The characteristic peaks at wave number 3321 cm^−1^ and wave number 2927 cm^−1^ are related to O-H stretching and C-H stretching, respectively. As compared with TC-TGase-LYS-0, films containing LYS showed higher intensity of these two characteristic peaks. Similar results were found in the literature [43].

### 3.6. Antimicrobial Properties of TC Films

Improper handling during processing and poor control during storage can lead to the growth of food spoilage and pathogenic microorganisms. Incorporation of antimicrobial agents into edible films is one of the strategies to inhibit the growth of microorganisms in foods [44]. Generally contaminated from the hands of processing workers, *Staphylococcus aureus* causes food poisoning through the production of staphylococcal enterotoxins. *Bacillus subtilis*, a spore-forming Gram-positive bacterium, is a common spoilage bacterium in various foods. Pathogenic *E. coli* contamination is associated with raw beef, raw milk, salad and drinking water and produces toxins and other metabolites that induce intestinal inflammatory responses in humans. *Pseudomonas fluorescens* secretes extracellular enzymes that are responsible for spoilage of meat and dairy products. Because of the regulatory limitations regarding conducting experiments with *Salmonella* strains in food plant microbiological laboratories, *Enterococcus faecium*, isolated from the human intestine, was used as a substitute. Studies have shown that *Enter. faecium* can be used to replace *Salmonella* in the evaluation of antibacterial ability in pepper, basil leaves, etc. [44,45].

The inhibitory activities of TC films against *S. aureus* LYC1659, *Enter. faecium* LYC 1513, *B. subtilis* BCRC 10029, *E. coli* LYC 1656 and *P. fluorescens* BCRC 11028 are shown in Table 4. The inhibition percentages increased as the amount of LYS increased. Furthermore, better inhibition was detected for Gram-positive bacteria (*S. aureus* LYC 1659, *Enter. faecium* LYC 1513, and *B. subtilis* BCRC 10029) than for Gram-negative bacteria (*E. coli* LYC 1656 and *P. fluorescens* BCRC 11028). LYS hydrolyzes the β-1,4 glycosidic bond between N-acetylaminoglucosamine and N-acetylmuramic acid of peptidoglycan in the cell walls of bacteria. LYS showed a weaker inhibitory effect on Gram-negative bacteria such as Pseudomonadaceae and Enterobacteriaceae [43], mainly because the cell walls of Gram-negative bacteria consist of more lipopolysaccharide and less peptidoglycan. On the other hand, the cell walls of Gram-positive bacteria are mainly composed of peptidoglycan. However, significant increases of inhibitory parentage against all five tested bacterial strains were detected for films with LYS as compared with films without LYS.

## 4. Conclusions

While TGase cross-linking and LYS addition levels did not markedly influence the visual appearance of PC films, LYS did significantly increase the opacity as compared with films without LYS. TGase cross-linking significantly reduced the water vapor permeability of TC films as compared with the control films. While the elongation at break decreased for films cross-linked with TGase, the tensile strength and Young’s modulus of the film gradually increased as the LYS content increased. LYS-incorporated TC films showed antimicrobial activity against *E. coli*, *Staphylococcus aureus*, *Enterococcus faecium*, *Bacillus subtilis* and *Pseudomonas fluorescens.* Collectively, TC films with improved physiochemical properties and antimicrobial activity have a good potential to serve as active food packaging materials. Furthermore, TGase can be used in the fabrication of protein-based edible films where the physiochemical properties need to be fortified.

## Figures and Tables

**Table 1:** Color attributes and opacity of transglutaminase (TGase) cross-linked tilapia collagen (TC) films incorporated with lysozyme (LYS).

Film Type	L*	a*	b*	WI	Opacity ^#^
Control film	95.27 ± 0.14 ^a^	−0.08 ± 0.01 ^a^	1.09 ± 0.02 ^a^	95.15 ± 0.13 ^a^	0.30 ± 0.01 ^b^
TC-TGase-LYS-0	92.96 ± 0.09 ^b^	−0.28 ± 0.01 ^c^	1.09 ± 0.02 ^a^	92.87 ± 0.09 ^b^	0.31 ± 0.01 ^b^
TC-TGase-LYS-2	95.29 ± 0.02 ^a^	−0.20 ± 0.02 ^b^	1.07 ± 0.05 ^a^	95.17 ± 0.03 ^a^	0.42 ± 0.01 ^a^
TC-TGase-LYS-4	95.33 ± 0.02 ^a^	−0.19 ± 0.03 ^b^	1.09 ± 0.07 ^a^	95.21 ± 0.03 ^a^	0.42 ± 0.02 ^a^
TC-TGase-LYS-6	95.34 ± 0.02 ^a^	−0.19 ± 0.01 ^b^	1.11 ± 0.01 ^a^	95.21 ± 0.02 ^a^	0.41 ± 0.01 ^a^
TC-TGase-LYS-8	95.37 ± 0.06 ^a^	−0.17 ± 0.07 ^b^	1.10 ± 0.04 ^a^	95.23 ± 0.05 ^a^	0.42 ± 0.01 ^a^

Data are presented as mean ± standard deviation, n = 3. Data in the same column with different lowercase superscript are significantly different (*p* < 0.05). ^#^ Opacity = ABS_600_/X; ABS_600_: the absorbance at 600 nm; X: the film thickness (mm).

**Table 2:** Thickness, moisture content, water solubility and water vapor permeability of transglutaminase (TGase) cross-linked tilapia collagen (TC) films incorporated with lysozyme (LYS).

Film Type	Thickness (mm)	Moisture Content (g/100 g)	Water Solubility (%)	Water Vapor Permeability (10^−10^ g m/m^2^ s Pa)
Control film	0.151 ± 0.004 ^b^	13.76 ± 0.15 ^a^	98.75 ± 1.38 ^a^	4.48 ± 0.19 ^a^
TC-TGase-LYS-0	0.151 ± 0.001 ^b^	12.74 ± 0.16 ^b^	98.87 ± 0.13 ^a^	1.97 ± 0.02 ^d^
TC-TGase-LYS-2	0.157 ± 0.002 ^ab^	12.08 ± 0.06 ^c^	96.59 ± 0.83 ^b^	3.33 ± 0.18 ^c^
TC-TGase-LYS-4	0.157 ± 0.005 ^ab^	12.12 ± 0.30 ^c^	96.02 ± 0.71 ^b^	3.32 ± 0.10 ^c^
TC-TGase-LYS-6	0.159 ± 0.004 ^a^	12.12 ± 0.17 ^c^	95.55 ± 0.48 ^b^	3.73 ± 0.27 ^b^
TC-TGase-LYS-8	0.159 ± 0.003 ^a^	12.02 ± 0.16 ^c^	95.18 ± 0.64 ^b^	3.84 ± 0.16 ^b^

Data are presented as mean ± standard deviation, n = 3. Data in the same column with different lowercase superscript are significantly different (*p* < 0.05).

**Table 3:** Mechanical properties of transglutaminase (TGase) cross-linked tilapia collagen (TC) films incorporated with lysozyme (LYS).

Film Type	Tensile Strength (MPa)	Elongation at Break (%)	Young’s Modulus (MPa)
Control film	1.49 ± 0.32 ^b^	13.54 ± 1.05 ^c^	109.51 ± 33.95 ^c^
TC-TGase-LYS-0	2.74 ± 0.42 ^a^	44.12 ± 2.62 ^a^	298.50 ± 44.98 ^b^
TC-TGase-LYS-2	2.87 ± 0.09 ^a^	40.02 ± 2.54 ^a^	305.71 ± 20.07 ^b^
TC-TGase-LYS-4	3.01 ± 0.39 ^a^	39.72 ± 3.51 ^a^	360.19 ± 24.96 ^a^
TC-TGase-LYS-6	3.02 ± 0.22 ^a^	38.58 ± 6.78 ^a^	363.63 ± 23.50 ^a^
TC-TGase-LYS-8	3.07 ± 0.46 ^a^	22.20 ± 3.93 ^b^	384.77 ± 23.14 ^a^

Data are presented as mean ± standard deviation, n = 3. Data in the same column with different lowercase superscript are significantly different (*p* < 0.05).

**Table 4:** The inhibitory percentage (%) of transglutaminase (TGase) cross-linked tilapia collagen (TC) films incorporated with lysozyme (LYS).

	Inhibitory Percentage (%)				
Film Type	*S. aureus*	*E. faecalis*	*B. subtilis*	*E. coli*	*P. fluorescens*
Control film	-	-	-	-	-
TC-TGase-LYS-0	2.80 ± 1.00 ^d^	4.03 ± 1.61 ^d^	4.86 ± 1.61 ^d^	2.14 ± 0.52 ^e^	3.62 ± 2.01 ^d^
TC-TGase-LYS-2	52.14 ± 4.53 ^c^	56.63 ± 3.02 ^c^	47.82 ± 3.37 ^c^	18.65 ± 1.23 ^d^	21.76 ± 6.79 ^c^
TC-TGase-LYS-4	57.38 ± 2.85 ^c^	57.89 ± 2.85 ^c^	60.65 ± 5.39 ^b^	23.90 ± 2.26 ^c^	36.85 ± 3.57 ^b^
TC-TGase-LYS-6	65.06 ± 1.81 ^b^	66.96 ± 4.42 ^b^	66.54 ± 3.97 ^b^	31.99 ± 1.07 ^b^	51.13 ± 6.70 ^a^
TC-TGase-LYS-8	75.07 ± 3.29 ^a^	75.01 ± 1.95 ^a^	80.77 ± 0.67 ^a^	39.07 ± 0.56 ^a^	58.57 ± 5.34 ^a^

Data are presented as mean ± standard deviation, n = 3. -: not detected. Data in the same column with different lowercase superscript are significantly different (*p* < 0.05).

## Data Availability

Data are contained within the article.
